# Novel RP-HPLC–DAD approach for simultaneous determination of chlorphenoxamine hydrochloride and caffeine with their related substances

**DOI:** 10.1186/s13065-024-01238-8

**Published:** 2024-07-19

**Authors:** Ahmed Ashraf, Norhan Badr ElDin, Yasmin Rostom, Badr A. El-Zeany, Ghada A. Sedik

**Affiliations:** Analytical Chemistry Department, Faculty of Pharmacy, Cairo University, Kasr El-Aini Street, Cairo, 11562 Egypt

**Keywords:** Caffeine, Chlorphenoxamine, Chlorphenoxamine N-Oxide, Green analytical chemistry, HPLC–DAD, Theophylline

## Abstract

Ensuring the quality control of active pharmaceutical ingredients is crucial for drug products being introduced into the market. Even for established drugs, it is necessary to maintain a cutting-edge impurity control system. To analyze caffeine and chlorphenoxamine hydrochloride in their binary mixture, as well as theophylline and chlorphenoxamine N-oxide as related substances, a reversed phase-high performance liquid chromatography combined with a diode array detector system was created. The chromatographic separation was conducted using a C18 X-select Waters® column. The mobile phase consisted of 20.0 mM potassium dihydrogen phosphate modified to pH 3 with o-phosphoric acid and methanol. A gradient elution program was adopted at a flow rate of 1.3 mL/min and detected at a wavelength of 222 nm. The present methodology demonstrates a concentration ranging from 2–60, 1–80, 0.5–20 to 0.4–20 µg/mL for chlorphenoxamine hydrochloride, caffeine, chlorphenoxamine N-Oxide and theophylline, respectively. Chlorphenoxamine N-Oxide, being an impurity of chlorphenoxamine was prepared by refluxing intact drug with 5% H_2_O_2_ for 24 h at 100 °C. One of the objectives of the analytical community is to promote the adoption of green analysis methods, which involve the development of environmentally friendly techniques. The levels of greenness and whiteness were evaluated using four specific tools: Eco-Scale System, GAPI, AGREE, and RGB tool. Furthermore, we have evaluated the greenness of the analytical method presented and compared its performance and greenness to that of the approach described in the literature. In this study, results from CPX and CAF analysis were compared to those obtained in a previous study. The result shows that there is no notable variation in precision and accuracy. The proposed method was validated in accordance with the requirements of ICH.

**Supplementary Information:**

The online version contains supplementary material available at 10.1186/s13065-024-01238-8.

## Introduction

**Fig. 1:**
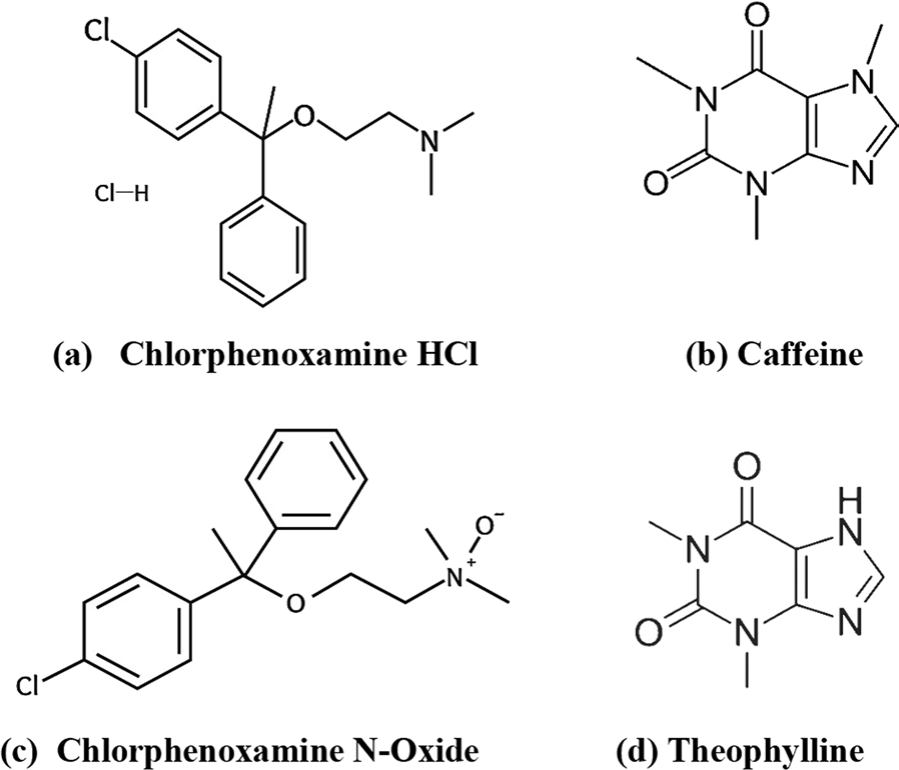
Chemical Structure of **a** Chlorphenoxamine HCl, **b** Caffeine, **c** Chlorphenoxamine N-Oxide and **d** Theophylline

An allergic reaction is triggered when the immune system responds to an external agent, such as pollen, specific types of food, insect bites or drugs [1, 2]. The range of symptoms associated with this condition spans from pruritus and respiratory irritation to urticaria and gastrointestinal distress [3]. Chlorphenoxamine hydrochloride (CPX); 2-[1-(4-chlorophenyl)-1-phenylethoxy]-N,N-dimethylethanamine[4]; Fig. 1, exhibits strong and sustained anti-histaminic and anticholinergic effects, making it a valuable treatment option for allergic conditions that are linked to cholinergic activity. Furthermore, and based on the Off-label use of FDA-approved medications, it is noteworthy to highlight the paramount significance of chlorphenoxamine hydrochloride in the therapeutic intervention of Middle East respiratory syndrome-related corona virus (MERS-COV), severe acute respiratory syndrome-related coronavirus (SARS-COV), Ebola virus (EBOV), and malaria owing to its G protein-coupled receptor (GPCR) antagonistic properties [5–7]. Caffeine (CAF); 1,3,7-trimethylpurine-2,6-dione[8]; Fig. 1, is always combined with CPX as its central stimulant effect nullifies the sedation action of CPX [9, 10]. Chlorphenoxamine N-Oxide (CPX N-Oxide); Fig. 1 is reported as CPX related substance [11, 12]. Theophylline (THP) is recognized as an impurity (related substance) of CAF, which results from the activity of cytochrome P450. This enzyme facilitates the N-7 demethylation of CAF, leading to formation of 1,3-dimethylxanthine (THP); Fig. 1 [13]. In contemporary times, the utilization of the Green analytical approach has become imperative to mitigate the depletion of hazardous solvents or reagents, thereby diminishing the impact of human activities on the environment. The Green Analytical Chemistry (GAC) endeavors to transform the field of analytical chemistry towards achieving environmental sustainability [14, 15]. This methodology employs environmentally conscious sample preparation techniques while also incorporating a concise time analysis. One of the objectives of utilizing the HPLC technique is to establish a state of balance between precision and accuracy of outcomes while minimizing potential environmental risks [16], in accordance with the guidelines set forth by GAC [17]. Various analytical techniques have been employed for the analysis of the proposed drugs in their dosage forms, including but not limited to HPLC [18, 19], supporting information Table S-1, spectrophotometry [20–22], differential pulse voltammetric [23], potentiometry [24] and thin layer chromatographic densitometric method [25]. These methods have been thoroughly researched and applied in the discipline of pharmaceutical analysis. There is no analytical method for analyzing CPX and CAF concurrently in the presence of CPX N-oxide and THP as related compounds. HPLC, being a widely employed analytical technique is utilized for both separation and quantification of drugs under investigation. The goal of this research is to create a low-cost, high-sensitivity HPLC method [26]. The assessment of the level of environmental friendliness was conducted through the utilization of various tools such as National Environmental Method Index [27], Analytical Eco-Scale [28], Green Analytical Procedure Index tool [29], Analytical Greenness Metric Approach and Software [30, 31].

## Experimental

### Instrumentation and software

An Agilent HPLC–DAD (1260 infinite II series) equipped with a quaternary pneumatic pumping system (model G1311C), an online degasser, a manual injector (20-μL) and a photodiode array detector (DAD) (model G1315D) was used in the experiment. In our study, we employed a Waters X select CSH® C_18_ (3.5 μm, 4.6 × 150 mm) C_18_ analytical column [32] To operate the apparatus, the Open LAB CDS ChemStation® software (version C.01.07) was used. Mobile phase and sample underwent filtration using 0.45-μm membrane filters and 0.22-μm disposable nylon syringe filters, respectively. Adjustment of pH was done by using pH meter (model 6300; Jenway, UK).

### Reagents

HPLC grade solvents were used, including potassium dihydrogen phosphate, methanol, hydrogen peroxide and o-phosphoric acid Sigma-Aldrich (Steinheim am Albuch, Germany).

### Samples

#### Pure samples

Pure standards of CPX and CAF were obtained from EIPICO Pharmaceuticals, 10th of Ramadan City, Egypt, with purities of 100.06 $\pm 0.47$% and 99.43 $\pm 0.60$% according to reported method [18]. THP was procured from Sigma-Aldrich (USA) with a certificate of purity of $\ge$ 99% [33]

#### Pharmaceutical formulation

Allergex® Caffeine tablets were produced by EIPICO Pharmaceuticals 10th of Ramadan City, Egypt. The tablet was supposed to have 20 mg CPX and 50 mg CAF. The source of acquisition was a local pharmacy in Egypt.

### Preparation of CPX N-Oxide solution

For CPX N-Oxide preparation, 100 mg of CPX was refluxed with 50 mL of 5% H_2_O_2_ for 24 h at 100 °C. The degradation process was monitored by TLC method throughout the disappearance of the spot corresponding to the intact drug and appearance of a new spot referring to the formation of the assumed degradation product using methanol: glacial acetic acid: water (5: 3: 2, by volume) as a creating system. The plates have been inspected using a UV light with a wavelength of 254 nm. After full degradation, a complete evaporation of the solution to get rid of H_2_O_2_ content, then, 10 mg of CPX N-Oxide was relocated into a 10 mL volumetric flask and subsequently diluted with methanol to attain a concentration of 1.0 mg/mL. The process of oxidative degradation was further monitored by HPLC method and mass spectrometry was used for the purpose of elucidating the structure of the degradation product.

### Procedure

#### Chromatographic conditions

The process of chromatographic separation was conducted within a temperature-controlled environment at a temperature of (25 ± 2 ºC), utilizing a reversed phase C_18_, X select column CSH. The gradient elution technique was carried out using a 20 mM potassium dihydrogen phosphate modified with o-phosphoric acid (pH 3.0 ± 0.2) as solvent (A) and methanol as solvent (B). The gradient has been established using three steps: initially, a ratio of 70:30 v/v was maintained for the first 0–1.5 min, followed by a transition to a ratio of 20:80 v/v through the subsequent 1.5–3 min. This ratio was then maintained until the end of the run, from 3 to 10 min. Prior to usage, the mobile phase was filtered through a 47 mm filter with a pore size of 0.45μm and was subjected to degassing for 15 min in an ultrasonic bath. With the detection of UV at 222 nm, a consistent flow rate of 1.3 mL/min was obtained. After 10 min, the mobile phase was gradually adjusted back to a ratio of 70:30 v/v, and then the mobile phase was run until a stable baseline was achieved and the pressure had reached a steady state.

#### Calibration curves construction

The working standard solutions of CPX, CAF, CPX N-Oxide and THP (100 µg/mL) were prepared through the process of dilution using the corresponding stock solutions (1.0 mg/mL). Then, the linearity of each analyte was assessed through serial dilution of working standard solutions, resulting in a concentration range of, (2–60 µg/mL) for CPX, (1–80 µg/mL) for CAF, (0.5–20 µg/mL) for CPX N-Oxide and (0.4–20 µg/mL) for THP with the used mobile phase as solvents. The solutions were subsequently passed through a 0.25 membrane and 20 µL of each solution were injected in triplets. The calibration curves were created by comparing peak areas to the corresponding concentrations under the previously specified chromatographic conditions.

### Application to pharmaceutical formulation

Ten tablets had been balanced before being ground into powder. An accurate portion of powder, equivalent to 20 mg CPX and 50 mg CAF, was placed precisely into a 100 mL volumetric flask. To improve the drug extraction process, 50 mL of methanol was introduced to the solution, which was then sonicated for 30 min. Methanol was added to the mark to complete the volume and the mixture was subsequently filtered using 0.25 µm membrane filter. Half milliliter of filtered solution was transferred into a 10 mL volumetric flask and subsequently filled to the mark with the mobile phase solution. The resulting concentrations and the percentage of recoveries (R%) of CPX and CAF were obtained using the regression equations. For application of the standard addition technique, Allergex Caffeine powder was spiked with standard CPX (10, 20, 40 mg) and CAF (25, 50, 100 mg). The procedure was completed as outlined in the analysis of the dosage form.

## Result and discussion

There has been a global push to promote environmentally sustainable analytical methods that generate non-hazardous waste and minimize the production of harmful materials. The utilization of HPLC methodology allows for the efficient separation and quantification of components within a brief timeframe, while using minimal quantities of environmentally friendly solvents [34, 35]. The focus of this study was to establish a precise and reliable analytical technique for detecting the studied analytes in their prescribed dosage form, as well as in the presence their corresponding related substances [36]. The suggested approach mainly differs from the reported methods [18] by identifying related substances such as Chlorphenoxamine N-Oxide and Theophylline [37, 38]. It is crucial to determine the presence of the caffeine impurity (Theophylline) as exceeding the permitted limit might lead to accelerated heart rate or other cardiac rhythm disturbances, particularly in individuals with heart conditions [39].

### Characterization of oxidative degradation product of CPX

**Fig. 2:**
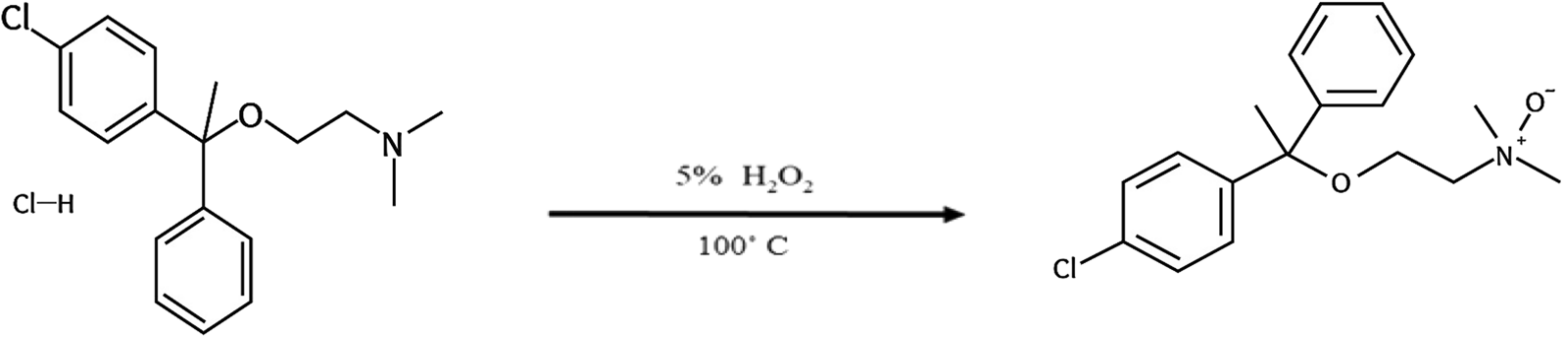
Oxidative degradation pathway of chlorphenoxamine HCl using 5% H_2_O_2_ for 24h at 100˚ C to obtain chlorphenoxamine N-Oxide

**Fig. 3:**
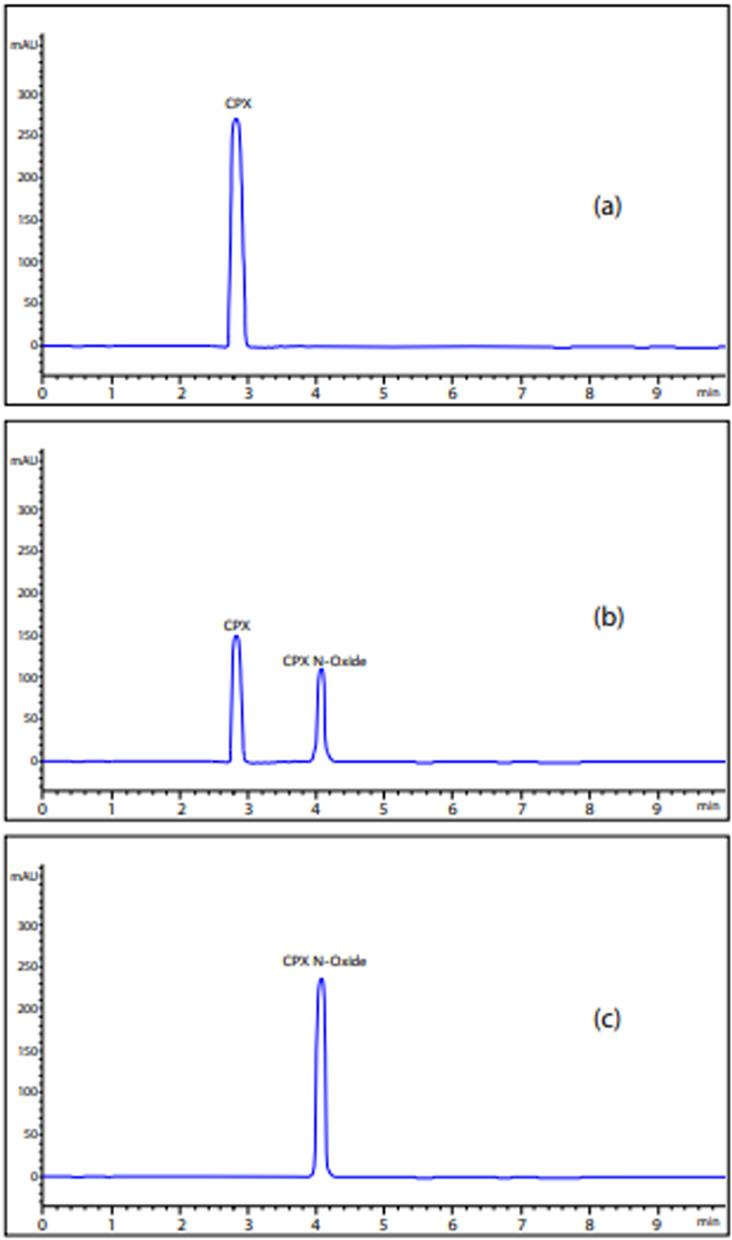
HPLC chromatograms during monitoring the CPX oxidative degradation at different time intervals starting with 60 µg/mL of CPX using methanol-20 mM potassium dihydrogen phosphate modified with o-phosphoric acid pH 3 (80/20, v/v) at 222 nm. **a** Zero time degradation of CPX. **b** After 12 hours degradation, peak of CPX N-Oxide has been observed. **c** After 24 degradation, peak of CPX has been disappered.

**Fig. 4:**
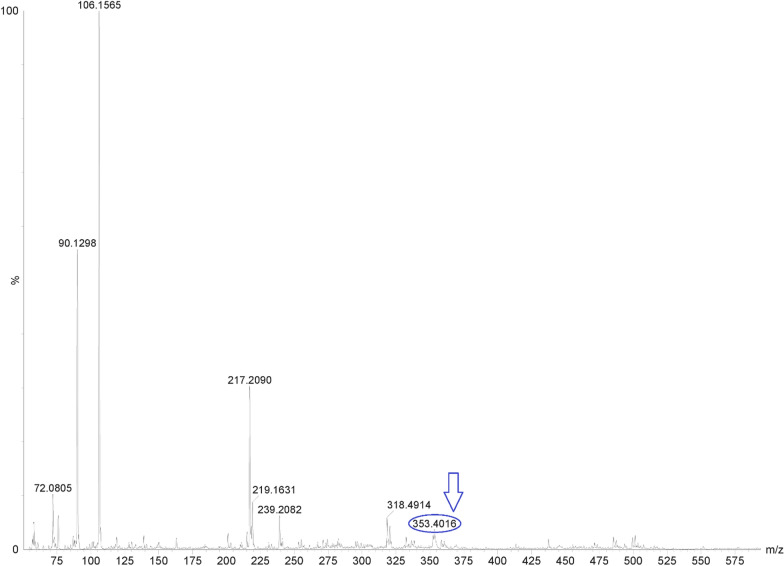
The Mass Spectrum at negative mode showing molecular ion peak [M-2H] = 353 m/z for CPX N-Oxide

The degradation procedure, as mentioned in "Preparation of CPX N-Oxide solution" Sect., was monitored through TLC technique. Various developing systems were employed to achieve optimal separation between CPX and its oxidative degradation product. An effective separation was achieved utilizing a developing system consisting of methanol, glacial acetic acid and water in a volumetric ratio of 5:3:2. The process of whole degradation was observed by tracking the removal of the drug point located at R_f_ 0.52 and the emergence of a single spot representing its oxidative degradation product at R_f_ 0.75, as illustrated in Fig. 2 and supporting information Figure S-1. This result was further confirmed by HPLC and Mass spectroscopy using a mobile phase consisting of methanol: potassium dihydrogen phosphate modified with o-phosphoric acid (pH 3) (80:20 v\v), starting with a concentration of 60 µg/mL of CPX, Fig. 3a. The obtained results indicate a reduction of approximately 50% in the peak of the drug (T_R_ = 2.9 min), followed by the presence of a novel peak at 4.1 min, Fig. 3b. Following a 24 h degradation process, it was observed that only the oxidative product peak remained, while the CPX peak had completely disappeared, as depicted in Fig. 3c. Moreover, the structure of CPX N-Oxide was illustrated by the mass spectrum where a molecular ion peak was revealed at m/z 353, Fig. 4, confirming the suggested pathway for oxidation as illustrated in Fig. 2.

### Method optimization and development

**Fig. 5:**
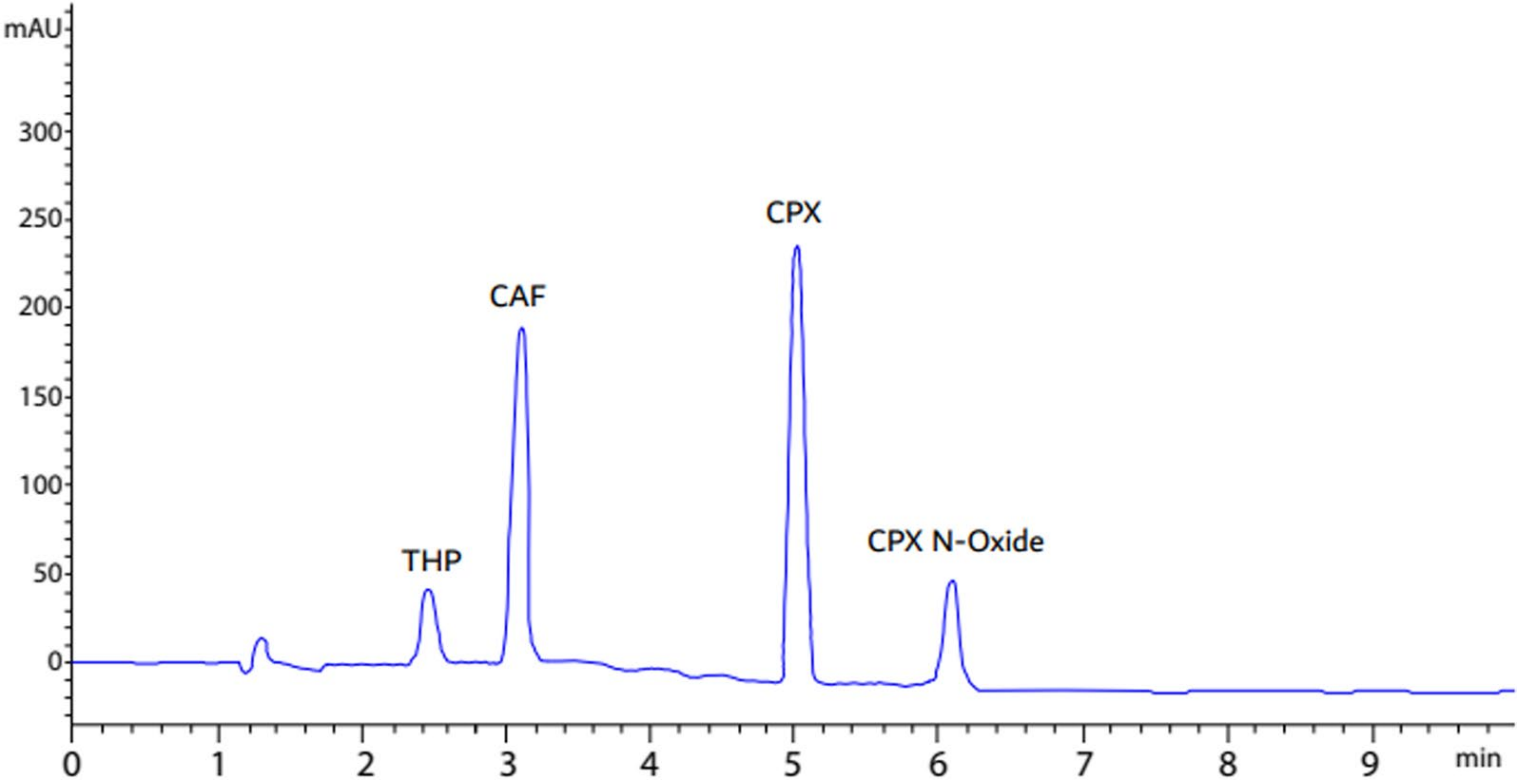
HPLC Chromatogram of standard, CPX (20 µg/mL) (t_R_ = 5.018), CAF (50 µg/mL) (t_R_ = 3.105), CPX N-Oxide (10 µg/mL) (t_R_ = 6.182) and THP (10 µg/mL) (t_R_ = 2.45) C_18_ X-select Waters® column (3.5 µm, 4.6 mm × 150 mm) and a gradient elution of solvent A (20 mM potassium di hydrogen phosphate adjusted to pH 3.0 with o-phosphoric acid) and solvent B (methanol) as a mobile phase at 222 nm

The objective of the HPLC method design was to utilize environmentally friendly chemicals and generate minimal hazardous waste while requiring a short analysis time [40]. This approach was designed to routinely determine combinations including CPX and CAF, as well as CPX N-Oxide and THP (as related compounds), without creating any environmental harm. Thus, two factors were considered: solvents safety and the minimization of the waste produced. Firstly, various combinations of ethanol/water and methanol/water solutions were examined in isocratic separation mode, however the resolution and separation results were unsatisfactory. Consequently, potassium dihydrogen phosphate buffer instead of water was considered. Various ratios of ethanol and phosphate buffer were tested at various pH levels (8 and 3), however, the results indicated inadequate separation. Furthermore, ethanol has a substantial disadvantage since the viscosity of ethanol/water mixtures is larger when compared to that of methanol/water for the same eluotropic intensity at ambient temperature [41]. High backpressures resulted from such high viscosity. As a result, methanol was preferred over ethanol. Different ratios of phosphate buffer and methanol were evaluated under various pH conditions via isocratic elution; however, this also resulted in low resolution. Reasonable results were procured while using methanol: potassium dihydrogen phosphate modified with o-phosphoric acid (pH 3). Even though raising buffer ratios CAF and THP revealed good separation, CPX and CPX N-Oxide showed bad separation. On the other hand, increasing methanol ratio CPX and CPX N-Oxide resulted in superior resolution, while CAF and THP exhibited inadequate separation. Thus, gradient elution system was the best solution to gain better resolution for all studied components. The drugs and their related substances were effectively separated and quantified using 20 mM potassium dihydrogen phosphate modified to pH 3 with o-phosphoric acid (solvent A) and methanol (solvent B), using a gradient mode of 0 min to1.5 min (70:30 v/v) and 1.5–3 min (20:80 v/v) with a flow rate of 1.3 mL/min. At those adjusted conditions, peaks of each component were obtained with a short retention time while maintaining satisfactory peak resolution. Furthermore, the suitability of the wavelength was evaluated by customizing the DAD detector at various wavelengths to determine the optimal conditions yielding the highest sensitivity for the analyzed components. The wavelengths 222 nm for CPX while 260 nm for CPX N-oxide, 272 nm for the detection of CAF and THP were detected. HPLC-chromatogram demonstrated that the retention time for CPX was 5.018 ± 0.1 min, for CAF, it was 3.105 ± 0.1 min. While for CPX-N-Oxide, it was 6.182 ± 0.1 min and for THP, it was 2.451 ± 0.1 min, Fig. 5. To verify that the suggested approach was valid, a system suitability analysis was carried out. Six duplicate injections of the reference solution at 100% each were given for the CPX, CAF, CPX N-Oxide and THP system suitability tests. Values of theoretical plates (≥ 2000), retention time (≤ 2.0%) and tailing factor (≤ 2.0) have been determined to be within the standard acceptability guidelines for the system suitability parameters. Supporting information Table S-2 presents the calculations of the system suitability parameters. The acquired results were verified using the USP Reference values [42].

### Method validation

The proposed method validation was evaluated according to the guidelines set forth by ICH [43].

#### Linearity and range

Six concentrations were analyzed in triplicates within specified ranges for each compound using RP-HPLC method. CPX was examined in the range of 2–60 µg/mL, CAF at 1–80 µg/mL, CPX N-Oxide at 0.5–20 µg/mL, and THP at 0.4–20 µg/mL under specific chromatographic conditions. There were linear correlations found between the average peak areas and corresponding concentrations as shown in Table 1.

**Table 1:** Regression and validation Parameters of the proposed HPLC -DAD method for determination of CPX and CAF and their related substances in pure form

Parameter	CPX	CAF	CPX N-Oxide	THP
Wavelength (nm)	222 nm	272 nm	260 nm	272 nm
Linearity				
range (µg/mL)	2–60	1–80	0.5–20	0.4–20
Intercept	14.567	12.694	11.445	5.4162
Slope	55.709	56.275	55.482	56.702
Correlation coefficient (r)	0.9995	0.9997	0.9996	0.9993
Precision(± %RSD)				
Repeatability^a^	0.89	0.847	2.02	1.17
Intermediate precision^b^	0.929	0.515	1.74	1.232
LOD^c^(µg/mL)	0.59	0.312	0.15	0.12
LOQ^c^(µg/mL)	1.79	0.945	0.43	0.33

^a, b^Intraday and interday precision [average three different concentrations of three replicate each (n = 9) within same day and repeated on 3 successive days, respectively]

^c^LOD and LOQ are calculated according to ICH, $\frac{3.3\times \,SD \,of \,residuals}{slope}$ and $\frac{10\times \,SD \,of \,residuals}{slope}$, respectively

#### Accuracy

Five pure samples at concentrations of (7.00, 20.00, 40.00, 60.00, and 80.00 µg/mL) for CPX and CAF, and (3.00, 6.00, 12.00, 14.00, and 17.00 µg/mL) for CPX N-Oxide and THP were examined in triplicate using RP-HPLC to verify the accuracy of the proposed methods. The percentage of recoveries were determined by utilizing the concentrations derived from the appropriate regression equations. The percentage recoveries from the suggested procedures were accurate, supporting information Table S-4.

#### Precision

Repeatability, three different concentrations were analyzed for CPX and CAF (5, 30, and 50 µg/mL) and for CPX N-Oxide and THP (2, 6, 10 µg/mL). The results can be found in Table 1, showing high precision with low values of percentage relative standard deviation (% RSD).

Inter-day precision was evaluated by replicating the same three concentrations of the drugs on three successive days. The RSD values demonstrated limited variability, confirming that the data was reasonably consistent Table 1.

#### Limit of detection and limit of quantification

The parameters LOD and LOQ were derived using the formulas LOD = 3.3 × SD/S and LOQ = 10 × SD/S, where SD represents the standard deviation of the response (peak area) and S is the slope of the calibration curve, Table 1.

#### Robustness

Robustness is the capacity of a system to withstand minor variations in method parameters without being affected. varying the pH by ± 0.2, adjusting the flow rate by ± 0.1 mL/min, and altering the wavelength by ± 2 nm. The response was monitored by observing changes in peak regions and calculating the %RSD. The results indicated that small intentional changes in the tested parameters did not affect the stability of the approaches, supporting information Table S-5.

### Analysis of the pharmaceutical formulation

The co-formulation of CPX and CAF in Allergex® Caffeine tablets was effectively evaluated using the proposed chromatographic method. The sample preparation was conducted utilizing a single step extraction utilizing methanol, indicating the removal of any intervention from the tablet additives. The validity of this method and a standard addition technique were presented in supporting information Table S-3. The favorable results and minimal sample manipulation procedures also direct focus towards the practicality of the suggested method as environmentally friendly procedures for monitoring the quality of the analytes.

### Statistical analysis

In this study, the results of CPX and CAF analysis were compared to those previously achieved by a reported one [18]. In supporting information Table S-4, it is found that the tabulated T and F values exceeded the calculated ones. The suggested and reported methods demonstrated no significant difference in terms of precision and accuracy.

### Assessment of the analytical method greenness profile.

The adoption of environmentally friendly analytical methods in the pharmaceutical industry, which replace hazardous solvent-based methods, has gained momentum due to the increasing concern for environmental protection [36, 44]. Within this context, assessing the environmental effects of various analytical methodologies in relation to their adherence to the guidelines of green analytical chemistry (GAC) has been believed a crucial undertaking [45]. Several evaluation tools for GAC have been introduced, commonly referred to as "green metrics." These metrics serve to provide a quantitative or qualitative assessment of the level of environmental sustainability associated with each analytical procedure [46, 47]. The evaluation was carried out using four different approaches.

- a. Analytical eco-scale system

Eco-Scale grading system based on penalty scores was devised as a partially quantitative way to measure how environmentally friendly the analytical methodology is [28]. The penalty scores are established for all parameters in the analytical process which influences the optimum analysis, such as the quantity of reagent, waste generated and energy exhaustion and are deducted from the ideal green technique base value of 100. The Eco-Scale grading is located in Table 2. The score of more than 75 attests to the method superior practices.

- b. Green analytical procedure index (GAPI)

**Table 2:** Greenness assessment of the proposed HPLC and reported HPLC method via different metrics

The proposed HPLC method				
Analytical Eco-scale		GAPI Tool	AGREE Tool	RGB Model
Reagent				
Methanol	6			
Phosphate Buffer	0			
5% H_2_O_2_	4			
Glacial acetic acid	4			
O-phosphoric acid	4			
Instruments				
Energy	1			
Occupational hazard	0			
Waste	6			
Total penalty points	25			
Total score	75			
The reported HPLC method				
Reagent				
Methanol	6			
Acetonitrile	6			
Triethylamine	8			
Instruments				
Energy	1			
Occupational Hazard	0			
Waste	6			
Total Penalty points	27			
Total score	73			

The combination of Eco-Scale and NEMI allows for the assessment of the total sustainability of analytical procedures using a new instrument called GAPI [29]. A unique pictogram with five pentagrams to evaluate, with the colors green, yellow and red signifying the low, medium and high ecological influence for each step. The GAPI tool provides information on sample preparation, the influence of reagents on health and safety, waste treatment and instrumentation. The GAPI pictogram is displayed in Table 2. Pictogram was created with more green-shaded portions (7) than red-shaded ones (4), indicating a more green-focused analytical approach than reported one.

- c. Analytical greenness metric approach and software (AGREE)

AGREE tool is the most recent consecrated universal metric tool [30] that evaluates the degree of greenness of the analytical approach based on the twelve principles, supporting information Figure S-2. The tool output is a clock—like graph, assessment score is displayed in the center of the circular pictogram, with colors from deep green to deep red. In this method the AGREE pictogram gave a score of 0.62, while for reported HPLC method was 0.52 Table 2.

- d. Assessment of whiteness of the proposed method versus published method

The novel Red Green Blue (RGB) 12 algorithm with three groups was acknowledged by Nowak et al. in 2021 [31]. Each group symbolizes a different color and contains certain criteria that assess crucial facets of the analytical method. Red group assesses the analytical effectiveness in terms of validation criteria, including scope of application, precision, accuracy and lowest LOD and LOQ. The well-known GAC principles are assigned to the green region and productivity factors including cost and time effectiveness, minimal practical requirements and operational simplicity are represented in the blue region. The RGB 12 model is displayed as an Excel spreadsheet that adhered to the WAC guidelines. The methodologies are plainly evaluated in accordance with the 12 WAC assumptions and the amount of sustainability as determined by the whiteness assessment is estimated. A well-balanced analytical procedure that is appropriate for the situation is referred to as "white" in the WAC approach. The recommended approach was looked at and critically contrasted with the reported approach Table 2.

## Conclusion

In this article, a newly developed and validated HPLC–DAD method was presented, offering several advantages for the simultaneous determination of CPX, CAF and their respective related substances. The method is not only fast and accurate, but also green and selective. Applying this method, it is possible to obtain highly sensitive and reliable results, which can be crucial for a variety of analytical applications. Also, the greenness method was evaluated using various assessment tools. These tools include Eco-Scale scoring, GAPI, and AGREE. The suggested method was found to have a minor environmental impact, as indicated by the evaluation results. The application of this technique for determining CPX and CAF in tablets has been found to be highly effective, as it is capable to accurately measure the compound without being affected by any excipients. The findings of this investigation shed light on the potential of CPX and CAF analysis as an alternative method for the quantification of the target compounds. To sum up, The HPLC method proposed is a significant and advanced approach for analyzing active pharmaceutical ingredients. It is noteworthy for its eco-friendliness, uncomplicated nature with minimal data manipulation, reproducibility, rapidity and precision.

### Supplementary Information

Additional file 1.

## Data Availability

The data used and/or analyzed during this study are available from the corresponding author on a reasonable request.

## Abbreviations

CPX: Chlorphenoxamine hydrochloride
CAF: Caffeine
DAD: Diode array detector
FDA: Food and drug administration
GAC: Green analytical chemistry
MERS-COV: Middle East respiratory syndrome-related corona virus
ICH: International conference of harmonization
SARS-COV: Severe acute respiratory syndrome-related corona virus
THP: Theophylline
