# Supplementary material for: Novel RP-HPLC–DAD approach for simultaneous determination of chlorphenoxamine hydrochloride and caffeine with their related substances
Source: BMC Chem. 2024 Jul 19;18(1):133. doi: 10.1186/s13065-024-01238-8 (PMC11264915; doi:10.1186/s13065-024-01238-8)
Supplement: Supplementary file 1 — Additional file 1. [file 13065_2024_1238_MOESM1_ESM.docx]

Supplementary Information

For

**Novel RP-HPLC-DAD Approach for Simultaneous Determination of Chlorphenoxamine Hydrochloride and Caffeine with Their Related Substances**


 Ahmed Ashraf *, Norhan Badr ElDin, Yasmin Rostom, Badr A. El-Zeany, Ghada A. Sedik
Analytical Chemistry Department, Faculty of Pharmacy, Cairo University, Kasr El-Aini Street, 11562 Cairo, Egypt
* Corresponding Author: Ahmed[.Ashraf@pharma.cu.edu.eg](mailto:.Ashraf@pharma.cu.edu.eg)


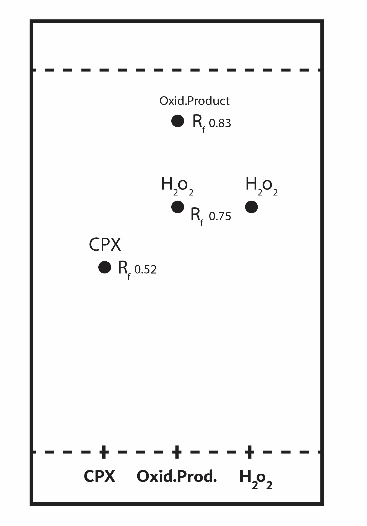


**Figure S-1: TLC plate showing CPX (R_f_ = 0.52) and Product (R_f_ = 0.78) using methanol,
 glacial acetic acid and water (5:3:2, by volume) as a developing system.**


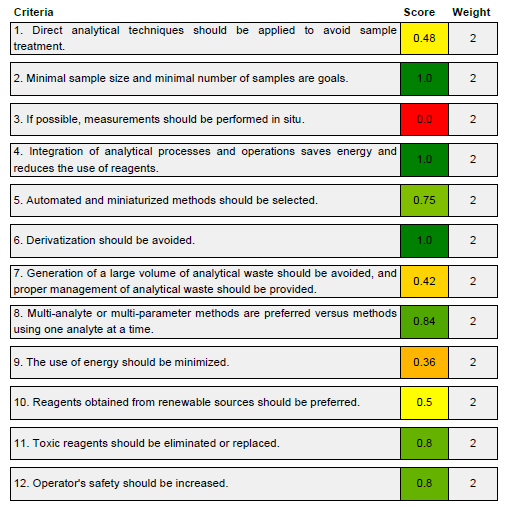


 **Figure S-2: The twelve principles of AGREE**

| **Column** | **Mobile Phase** | **Flow Rate (mL/min)** | **Detection** | **Application** | **Linearity range (µg/mL)** |
| --- | --- | --- | --- | --- | --- |
| **C_18_ ^a^** | 20 mM potassium dihydrogen phosphate modified with o-phosphoric acid pH 3 (solvent A) and methanol (solvent B) | 1.3 | UV detection at 222 nm. | Separation of four compounds in Dosage form | CPX (2-60), CAF (1-80), CPX N-Oxide (0.4-20), and THP (0.5-20) |
| **C_8_ ^b^** | ethyl acetate/methanol (50:50 v/v) /triethylamine pH 9 | 1 | UV detection at 254 nm. | Separation of three compounds Dosage form | CPH (37.5-725), CAFF (0.75- 48), and CTH (0.1- 40) |
| **Polaris SI ^c^** | Ethanol | 0.4 | UV detection at 220.4, 270.4, and 276.4 nm for CPH, CAFF, CTH, respectively. | Separation of three compounds Dosage form | CPH (1-70), CAFF (1.5- 35), and CTH (1- 40) |

**Table S-1: Comparative study between the proposed HPLC-DAD method and the reported HPLC methods**

**^a^** The proposed method
**^b^** The reported method [18]
**^c^** The published method [19]

| **Parameter** | **Obtained values** | | | | | | | **Reference values [42]** |
| --- | --- | --- | --- | --- | --- | --- | --- | --- |
|  | **THP** | **CAF** | | **CPX** | | **CPX N-Oxide** | |  |
| **Retention time (t _R_) ±0.03** | 2.451 | 3.105 | | 5.018 | | 6.182 | | **---------** |
| **Tailing factor (T)** | 1.25 | 1 | | 1 | | 1.25 | | **T**$\boldsymbol{\leq}$ **2** |
| **Resolution factor (R_s_)** | 2.97 9.57 6.5 | | | | | | | **R_s_ > 2** |
| **Selectivity factor (α)** | 1.56 2.06 1.31 | | | | | | | **α > 1** |
| **Number of theoretical plates (N)** | 2403 | | 2678 | | 15737 | | 2403 | **N >2000** |
| **HETP** | 6.24x10^-3^ | | 5.6x10^-3^ | | 9.5x10^-4^ | | 6.24x10^-3^ | **The smaller the value, the higher the column efficiency** |

**Table S-2: System suitability parameters of the proposed HPLC-DAD method for
 determination of CPX, CAF and their respective related substances.**

**^a^** $Selectivity (ꭤ) = K2‛/K1‛$ calculated for each two successive peaks.
**^b^** $Resolution (Rs) = 2 ( tRB -tR ) / WB+WA$ calculated for each two successive peaks.
**^c^** $Tailing Factor (T) = W0.05 / 2 ƒ$
**^e^** $Column efficiency (N) = \frac{16{tR}^{2}}{W^{2}}$**^d^** $Height equivalent to theoretical plate ( cm/plate) H= L/N$

**Table S-3: HPLC–DAD methods for the determination of CPX and CAF in Allergex Caffeine tablets® and application of standard addition technique**.

| **Allergex Caffeine^®^ tablets**  **Labelled to contain 20 mg CPX, 50 mg CAF** | | **Found±**  **%RSD ^a^** | **Standard Addition** | | |
| --- | --- | --- | --- | --- | --- |
|  |  |  | **Claimed amount  taken.**  **(µg/mL)** | **Standard added (µg/mL)** | **Recovery of standard added %**  **± %RSD ^b^** |
| **Batch number 2009308** | **CPX** | 100.80  ±1.193 | 10.00  10.00  10.00 | 5.00  10.00  20.00 | 99.44 ± 1.213  99.54 ± 0.985  101.09 ± 0.892 |
|  | **CAF** | 100.95  ±0.659 | 25.00  25.00  25.00 | 12.50  25.00  50.00 | 101.84 ± 1.145  101.93 ± 0.735  102.14 ± 0.820 |

**^a^** Average of five determinations **^b^** Average of three experiments.

**Table S-4: Accuracy (Mean ± SD) average of five different concentrations of three replicates (n = 15)**

| **Parameter** | **CPX** | **CAF** | **CPX N-Oxide** | **THP** |
| --- | --- | --- | --- | --- |
| **First level** (µg/mL)  (3 for CPX, CAF, and 1 for CPX N-Oxide, THP) | 99.56 ± 1.23 | 100.78 ± 1.15 | 99.85 ± 0.98 | 100.34 ± 0.95 |
| **Second level** (µg/mL)  (14 for CPX, CAF, and 3 for CPX N-Oxide, THP) | 98.44 ± 1.14 | 99.1 ± 0.95 | 100.5 ± 0.85 | 98.3 ± 0.94 |
| **Third level** (µg/mL)  (25 for CPX, CAF, and 4 for CPX N-Oxide, THP) | 101.2 ± 1.54 | 100.5 ± 1.34 | 101.6 ± 0.65 | 98.2 ± 0.52 |
| **Fourth level** (µg/mL)  (45 for CPX, CAF, and 7 for CPX N-Oxide, THP) | 99.6 ± 1.84 | 99.5 ± 0.89 | 98.7 ± 0.53 | 99.4 ± 0.59 |
| **Fifth level** (µg/mL)  (55 for CPX, CAF, and 14 for CPX N-Oxide, THP) | 100.25 ± 1.155 | 101.77 ± 1.46 | 99.05 ± 0.54 | 98.41 ± 0.55 |
| **Mean ± SD** | 99.81 ± 1.381 | 100.33 ± 1.158 | 99.94 ± 0.71 | 98.93 ± 0.71 |

| **Drug** | | **Robustness Parameter** | | | **T_R_** | **Tailing Factor** |
| --- | --- | --- | --- | --- | --- | --- |
| **CPX** |  | | Flow rate | 1.3 +0.1 mL/ min | 5.12 | 1.1 |
|  |  |  |  | 1.3 - 0.1 mL/ min | 5.08 | 1.25 |
|  |  |  | pH values | 3 + 0.2 units | 4.91 | 0.97 |
|  |  |  |  | 3 – 0.2 unit | 5.3 | 1.05 |
|  |  |  | Wavelength | 222 +2 nm | 4.8 | 1.2 |
|  |  |  |  | 222 –2 nm | 5.2 | 1.3 |
| **CAF** |  | | Flow rate | 1.3 +0.1 mL/ min | 3.2 | 1.3 |
|  |  |  |  | 1.3 - 0.1 mL/ min | 2.9 | 1.1 |
|  |  | | pH values | 3 + 0.2 units | 3.1 | 1.24 |
|  |  |  |  | 3 – 0.2 unit | 3.3 | 1.27 |
|  |  | | Wavelength | 222 +2 nm | 2.87 | 0.98 |
|  |  |  |  | 222 –2 nm | 2.95 | 1.5 |
| **CPX N-Oxide** |  | | Flow rate | 1.3 +0.1 mL/ min | 6.2 | 1.3 |
|  |  |  |  | 1.3 - 0.1 mL/ min | 5.87 | 1.75 |
|  |  | | pH values | 3 + 0.2 units | 6.1 | 1.52 |
|  |  |  |  | 3 – 0.2 unit | 6.3 | 1.46 |
|  |  | | Wavelength | 222 +2 nm | 6.12 | 1.66 |
|  |  |  |  | 222 –2 nm | 5.86 | 1.25 |
| **THP** |  | | Flow rate | 1.3 +0.1 mL/ min | 2.4 | 1.4 |
|  |  |  |  | 1.3 - 0.1 mL/ min | 2.61 | 1.1 |
|  |  | | pH values | 3 + 0.2 units | 2.1 | 1.63 |
|  |  |  |  | 3 – 0.2 unit | 1.93 | 1.34 |
|  |  | | Wavelength | 222 +2 nm | 2.21 | 1.27 |
|  |  |  |  | 222 –2 nm | 2.45 | 1.33 |

**Table S-5: Robustness** **is assessed by applying small changes in scanning wavelength (± 2 nm), flow rate (± 0.1 mL/min) and buffer pH (± 0.2)**

**Table S-6: Statistical comparison for the results obtained by the proposed HPLC methods
 and the reported method for CPX and CAF in its pure powdered form.**

| Item | CPX | | CAF | |
| --- | --- | --- | --- | --- |
|  | HPLC method | Reported method ^a^[18] | HPLC method | Reported method ^a^[18] |
| Mean | 100.207 | 100.06 | 99.81 | 99.43 |
| S.D. | 0.624 | 0.47 | 0.722 | 0.60 |
| Variance | 0.787 | 0.22 | 0.521 | 0.36 |
| n | 6 | 6 | 6 | 6 |
| Student's t-test ^b^(2.23) | 0.908 |  | 1.206 |  |
| F value ^b^ (5.05) | 3.58 |  | 1.45 |  |

**^a^** HPLC method with mobile phase consisting of ethyl acetate/methanol (50:50 v/v) /triethylamine pH 9 using Column: Shimpack NP-Sil. (150mm x 6mm i.d.,5μm) , (Shimadzu, Japan). , isocratic elution, and UV detection at 254 nm.
**^b^** values in parentheses are the corresponding theoretical values for t and F at P = 0.05
